# MRI findings from the trial of early aggressive therapy (TREAT) study

**DOI:** 10.1186/1546-0096-10-S1-A36

**Published:** 2012-07-13

**Authors:** Mahesh Thapa, Steven J Spalding, Philip J Hashkes, Sarah Ringold, Andrew S Zeft, Robert P Sundel, Randolph K Otto, Edward H Giannini, Daniel J Lovell, Carol A Wallace

**Affiliations:** 1 Children's Hospital Regional Medical, Seattle, WA, USA; 2 Childrens Hospital Medical Center, Boston, MA, USA; 3 Cincinnati Children's Hospital Medical Center,Cincinnati, OH, USA; 4 Cleveland Clinic, Cleveland, OH, USA; 5 Seattle Children's & University of Washington, Seattle, WA, USA; 6 Shaare Zedek Medical Center, Jerusalem, Israel; 7 University of Utah, Salt Lake City, UT, USA

## Purpose

MRI evaluation can be a helpful adjunct to clinical assessment of disease states in adult RA but has not been extensively investigated in children with JIA. MRI is a sensitive examination available to detect active synovial inflammation and can identify bone edema, known to be an important predictor for future joint erosions. By evaluating for such abnormalities, MRI could help differentiate children with active JIA from those with inactive disease (ID). The objectives were to determine if MRI with IV gadolinium (gad) (i) correlates with the clinical state of disease activity and (ii) can biologically confirm the state of clinically ID in children with JIA treated with early aggressive therapy.

## Methods

TREAT was a 12 mo randomized, double blind, multi-centered clinical trial in 85 participants aged 2 to 16 yrs with polyarticular or extended oligoarticular JIA of ≤ 12 mos duration. Participants were randomized 1:1 into 1 of 2 aggressive treatment arms: Arm 1- MTX 0.5 mg/kg/wk SQ (40 mg max), plus etanercept 0.8 mg/kg/wk (50 mg max), plus prednisone 0.5 mg/kg/d (60 mg max) tapered to 0 by 17 wks, or Arm 2 - MTX (same dose) plus etanercept and prednisolone placebo. The primary outcome was achievement of ID by 6 mos. At 4 mos participants not achieving an ACR Pediatric 70 were treated with open label Arm 1 meds. A subset of participants ≥ age 6 with knee synovitis underwent MRI with IV gad (0.1mg/kg) of 1 affected knee at baseline and after 6 mos of therapy. MRI pulse sequences included: single plane of fat-saturated fluid sensitive, single plane of T1 fat-saturated pre IV gad, 2 planes of T1 fat-saturated post IV gad, single plane of 3D spoiled gradient, and a single plane of Proton Density. Blinded MRIs were read independently by 2 radiologists.

## Results

11 participants were recruited into the MRI sub-study. At baseline, physician examination was concordant with MRI abnormalities of synovitis in 11 of 11 and effusion in 10 of 11. Of these, 3 of 11 had enlarged lymph nodes in the popliteal fossa, and 1 each had: prepatellar soft tissue edema, a multiseptated baker’s cyst with partial rupture, and bony edema; none had cartilage loss or erosions. There were 10 6-mo follow-up MRIs of which 6 showed complete resolution of abnormalities, including enlarged lymph nodes and prepatellar soft tissue edema. Four of these 6 participants were in Arm 1 and 2 were in Arm 2. The remaining 4 follow up MRIs (3 in Arm 1, 1 in Arm 2) showed improvements in the amount of synovitis and effusion, but new bony edema in 2 of these. At 6 mos, there was discordance between clinical exam and MRI findings in 40% of participants: 2 had clinical findings of knee swelling, but no abnormalities on MRI, and 2 had a normal clinical exam but abnormal MRI findings of synovitis and effusion. None of those with abnormal MRIs at follow up had achieved clinical ID.

## Conclusion

Joint damage/erosions were rare in this patient cohort. MRI evaluation may be a helpful adjunct for documentation of the clinical state of inactive disease in patients with JIA and assist in the correct classification of disease state.

## Disclosure

Mahesh Thapa: None; Steven J. Spalding: None; Philip J. Hashkes: None; Sarah Ringold: None; Andrew S. Zeft: None; Robert P. Sundel: None; Randolph K. Otto: None; Edward H. Giannini: None; Daniel J. Lovell: Abbott Laboratories, 9, Amgen Inc., 5, Bristol-Myers Squibb, 9, Centocor, Inc., 9, Hoffmann-La Roche, Inc., 9, Novartis Pharmaceuticals Corporation, 9, Regeneron Pharmaceuticals, Inc., 9, UBC, 9; Carol A. Wallace: Amgen Inc., 2.

